# Supplementary material for: A proteomics approach to isolating neuropilin-dependent α5 integrin trafficking pathways: neuropilin 1 and 2 co-traffic α5 integrin through endosomal p120RasGAP to promote polarised fibronectin fibrillogenesis in endothelial cells
Source: Commun Biol. 2024 May 24;7:629. doi: 10.1038/s42003-024-06320-4 (PMC11126613; doi:10.1038/s42003-024-06320-4)
Supplement: Supplementary file 2 — Supplementary Figs. [file 42003_2024_6320_MOESM2_ESM.pdf]

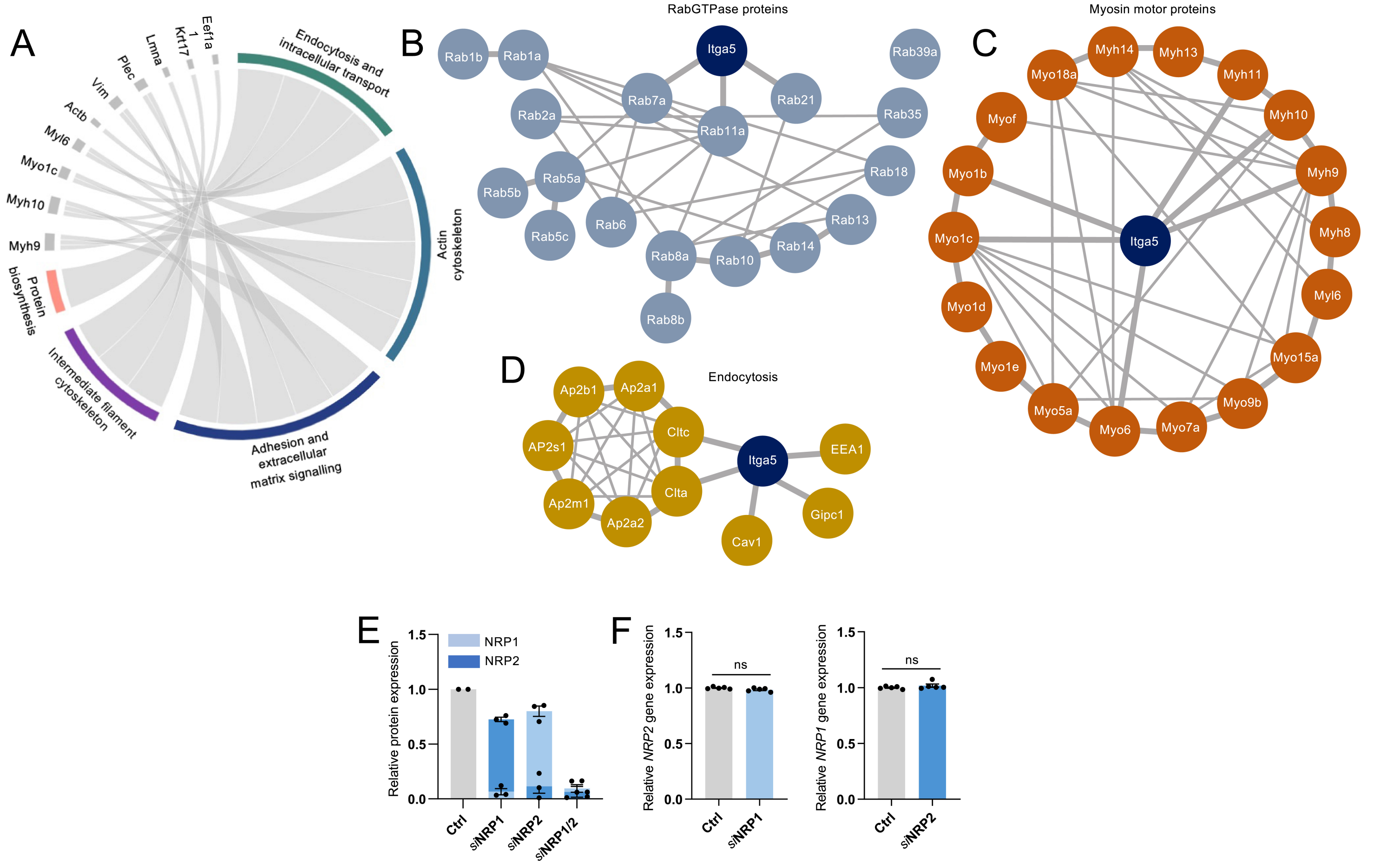

Suppl. Figure 1: A) Chord plot showing pathways associated with the top 10 proteins (sorted by intensity score) associating with α5 integrin. B-D) STRING interaction networks identifying associations between α5 integrin and RabGTPase proteins (B), myosin motor proteins (C) and Endocytic trafficking-associated proteins (D). E) Supporting densitometric analysis of Figure 1O demonstrating siRNA-mediated depletion of NRP1 and NRP2, (N = 3 independent experiments). F) NRP1 and NRP2 gene expression in siNRP2 and siNRP1 ECs respectively, relative to GAPDH, and shown alongside Ctrl samples, (N = 2 independent biological experiments (n = 5 total technical replicates), Student's t-test, ns = non-significant.

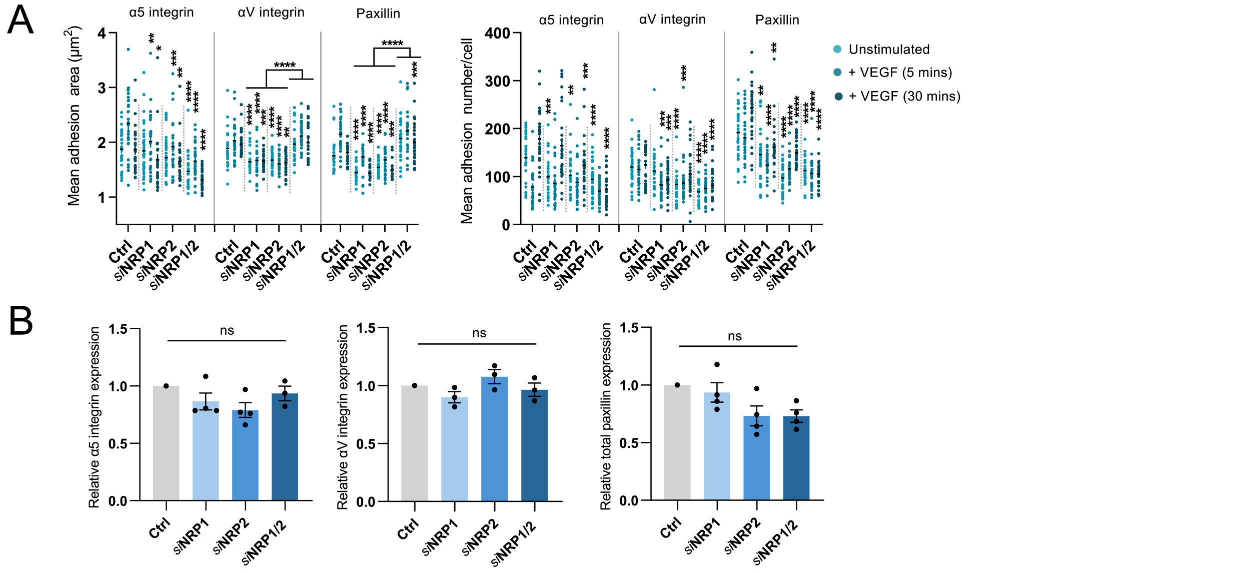

Suppl. Figure 2: A) Analysis of α5 integrin, αV integrin and paxillin+ adhesion size (μM2) (left) and number (right) ± 5 or 30 mins VEGF165 stimulation in Ctrl, siNRP1, siNRP2 and siNRP1/2 ECs, (n = 30 cells from 3 independent biological replicates), one-way ANOVA + Post Hoc multiple comparisons tests, **p < 0.01, ***p < 0.001, ****p < 0.0001). B) Densitometric analysis of total expression levels of α5 integrin, αV integrin and paxillin by Western blotting as shown in Figure 2G, (N = 3 independent experiments), one-way ANOVA + Post Hoc multiple comparisons tests, ns = non-significant.

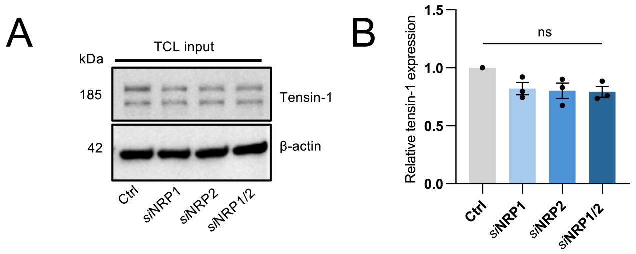

Suppl. Figure 3: A) Total cell lysate input showing expression levels of tensin-1 by Western blotting in siRNA-depleted ECs. B) Supporting densitometric analysis of A), (N = 3 independent experiments), one-way ANOVA + Post Hoc multiple comparisons tests, ns = non-significant.

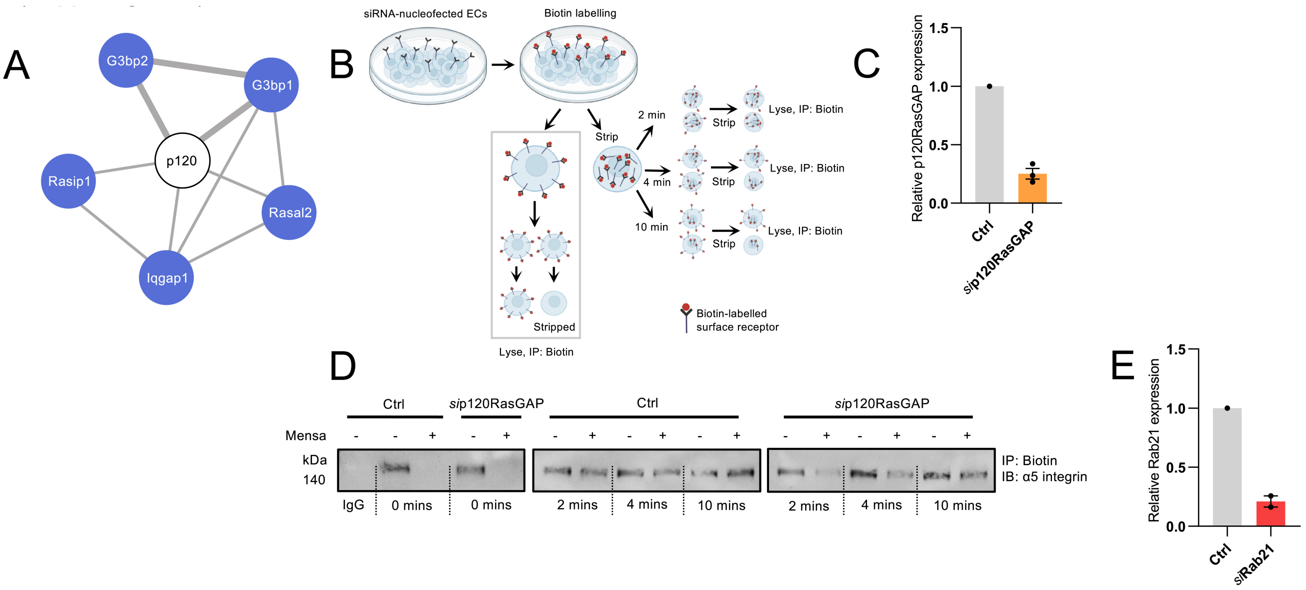

Suppl. Figure 4: A) Recycling assay schematic. Surface proteins were biotin labelled prior to incubation at 37°C to stimulate receptor internalisation. Following MESNA stripping, internalised proteins were allowed to recycle for the indicated timepoints at 37°C alongside a MESNA stripped control. EC lysates were immunoprecipitated against biotin to detect the recycled fraction of total α5 integrin by SDS-PAGE and Western blotting. B-C) Quantification of siRNA-depletion of p120RasGAP and Rab21 from total cell lysates, (N = 3, N = 2 independent experiments respectively). B) Created with BioRender.com.

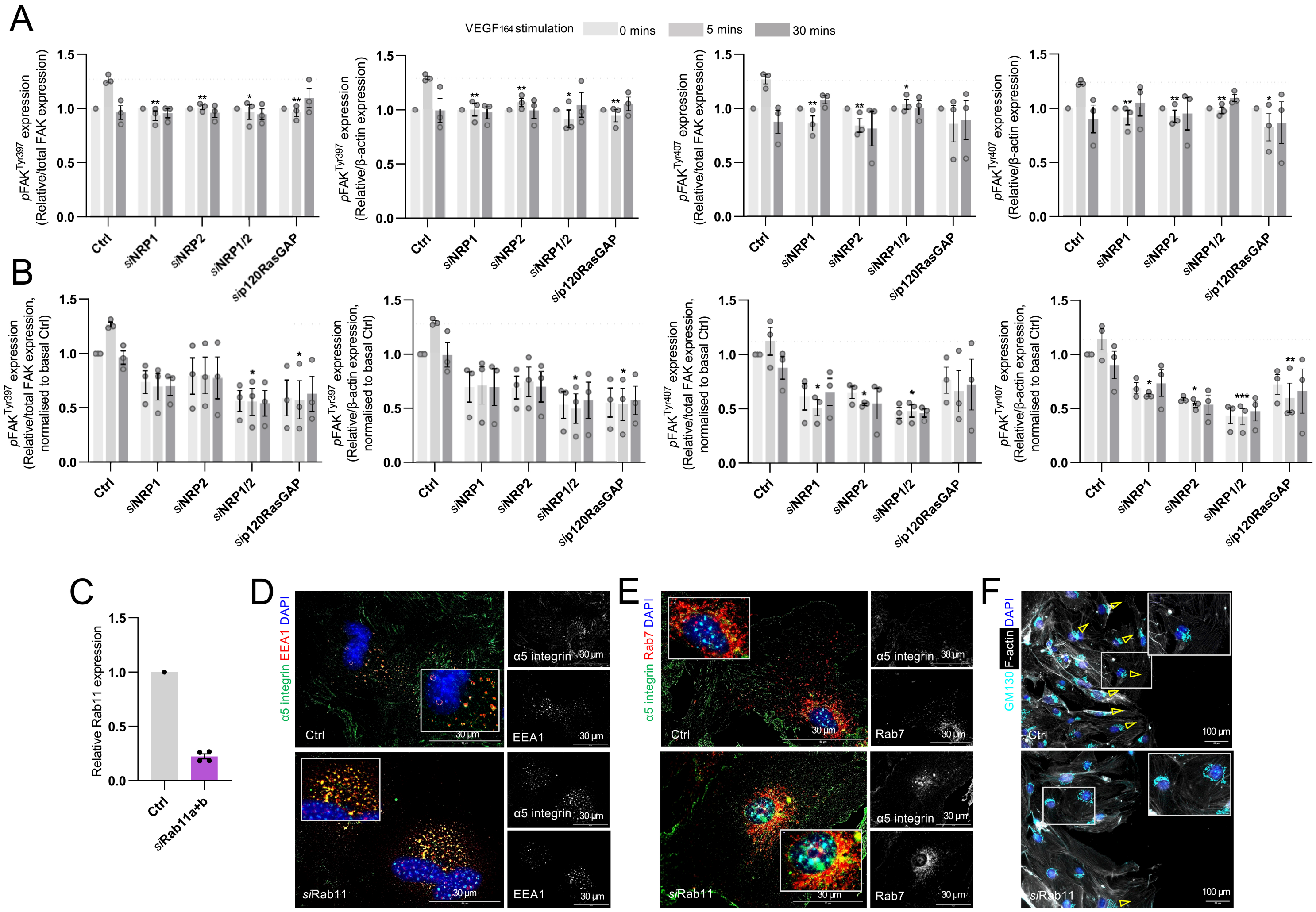

Suppl. Figure 5: A) Densitometric quantification of FAK phosphorylation at Try397 and Tyr407 residues relative to total FAK or β-actin expression in Ctrl and siRNA-depleted ECs, (N = 3 independent experiments), one-way ANOVA + Post Hoc multiple comparisons tests, *p < 0.05, **p < 0.01. B) Densitometric quantification of FAK phosphorylation at Try397 and Tyr407 residues relative to total FAK or β-actin expression in Ctrl and siRNA-depleted ECs, normalised to Ctrl basal conditions, (N = 3 independent experiments), one-way ANOVA + Post Hoc multiple comparisons tests, *p < 0.05, **p < 0.01, ***p < 0.001. C) Quantification of siRNA-depletion of Rab11 from total cell lysates, N = 4. D) Representative confocal microscopy images showing colocalisation between α5 integrin and EEA1 in Ctrl and siRab11 ECs fixed at 180 minutes. E) Representative confocal microscopy images showing colocalisation between α5 integrin and Rab7 in Ctrl and siRab11 ECs fixed at 180 minutes. F) Representative confocal microscopy images showing GM130+ Golgi-body positioning and F-actin protrusions in Ctrl and siRab11 ECs at the scratch-wound edge. Arrows indicate correctly positioned Golgi-bodies.

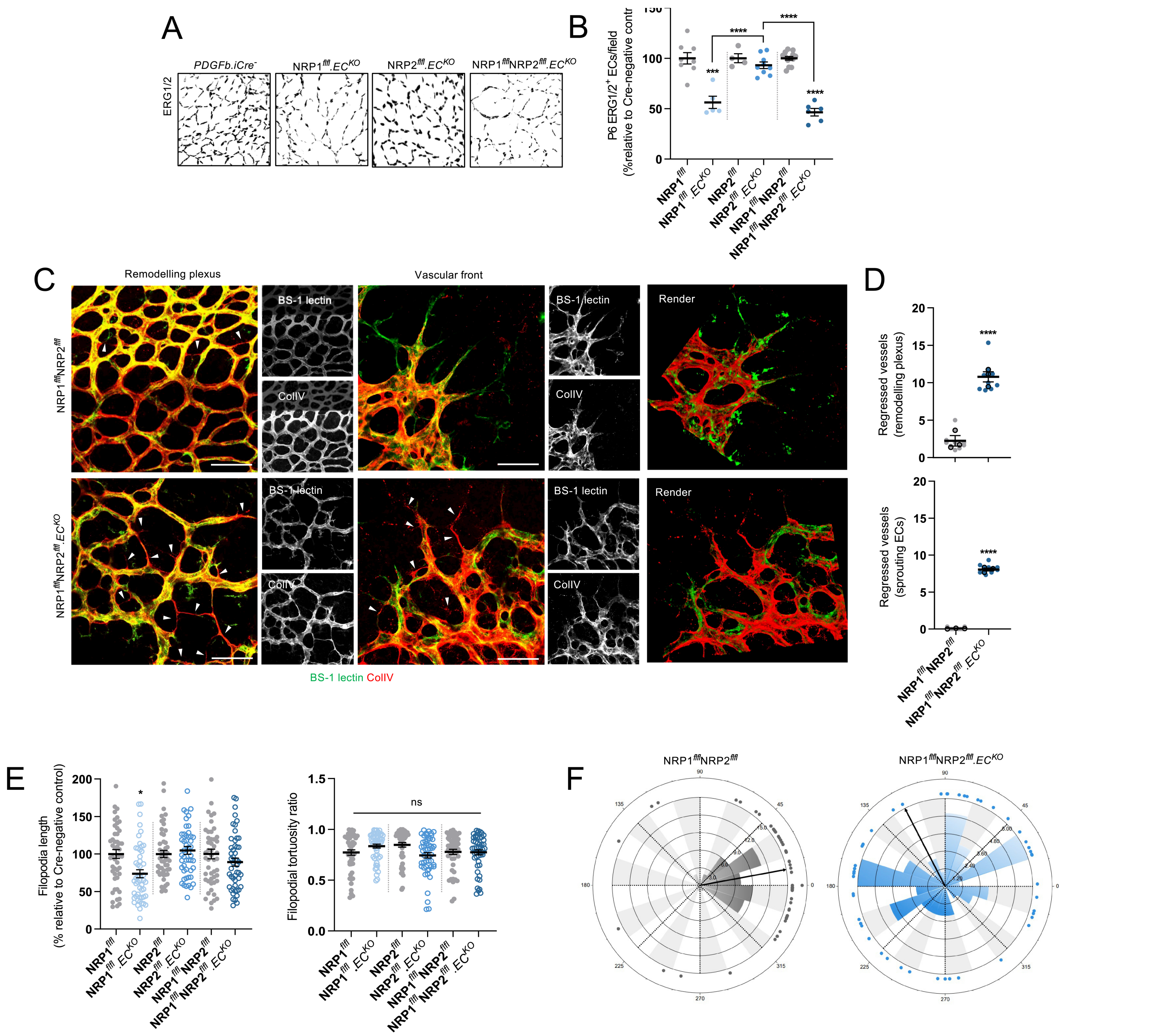

Suppl. Figure 6: A) Representative confocal microscopy images showing ERG1/2+ ECs in Pdgfb-iCreERT2-negative, NRP1flfl.ECKO, NRP2flfl.ECKO and NRP1flflNRP2flfl.ECKO retinas harvested at P6. B) Quantification of ERG1/2+ EC density shown as relative percentages of respective littermate control animals, (N ≥ 2 independent experiments (n ≥ 4 retinas)), Student's t-tests/one-way ANOVA + Post Hoc multiple comparisons tests, ***p < 0.001, ****p < 0.0001. C) Representative confocal microscopy images showing BS-1 lectin- collagen IV+ regressed vessels in Pdgfb-iCreERT2 negative and NRP1flflNRP2flfl.ECKO animals. D) Quantification of raw vessel regression, (N = 3 independent experiments (n ≥ 8 retinas)) Student's t-tests, ****p < 0.0001. E) Left panel: quantification of filopodial length (shown as relative percentages of respective littermate control animals). Right panel: filopodial tortuosity (shown as ratios) in Pdgfb-iCreERT2-negative, NRP1flfl.ECKO, NRP2flfl.ECKO and NRP1flflNRP2flfl.ECKO animals, (n = 5 retinas), Student's t-tests/one-way ANOVA + Post Hoc multiple comparisons tests, *p < 0.05, ns = non-significant. F) Individual polar plots showing Golgi-body orientation in Pdgfb-iCreERT2 negative and NRP1flflNRP2flfl.ECKO animals (n = 50 ECs).

Figure 1a

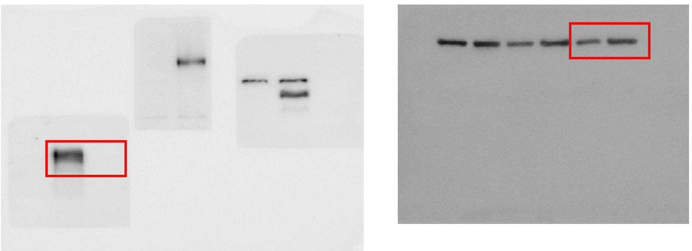

Figure 1f

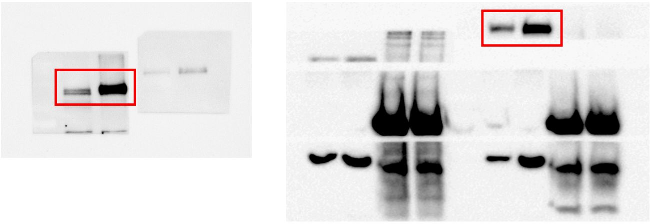

Figure 1g

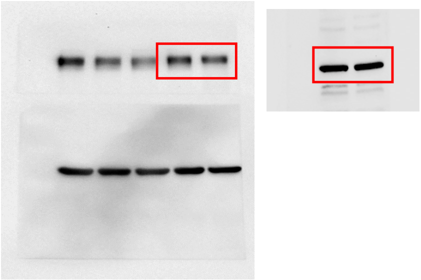

Figure 1o

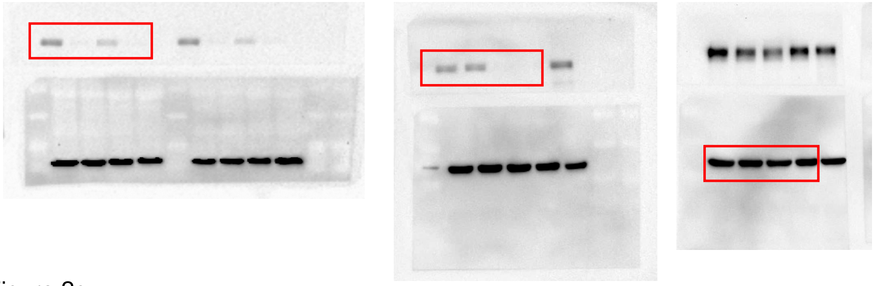

Figure 2g

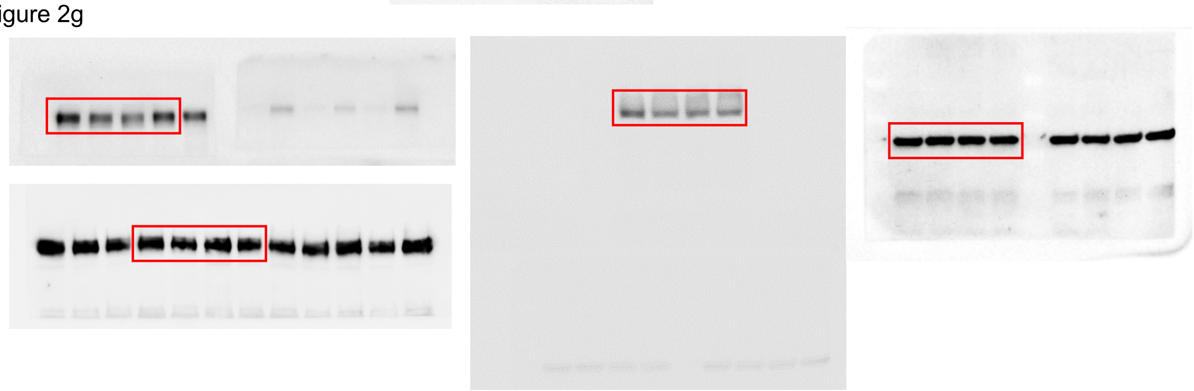

Figure 4c

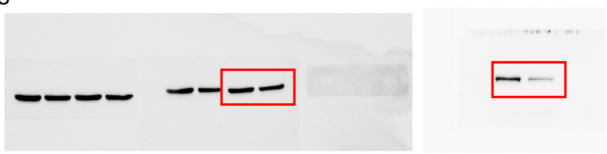

Figure 4i

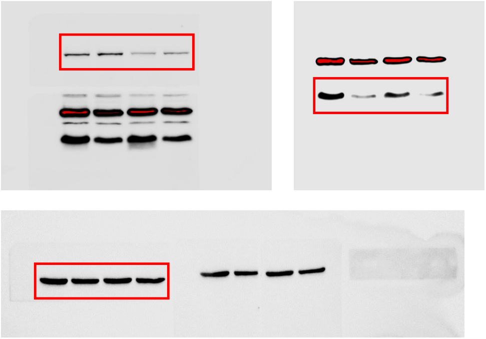

Suppl. Figure 7: Uncropped/raw gel images associated with Figures 1, 2 and 4. Red boxes indicate region of cropped image.

Figure 5a

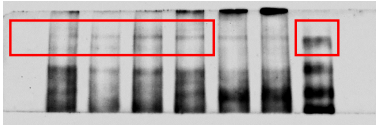

Figure 5b

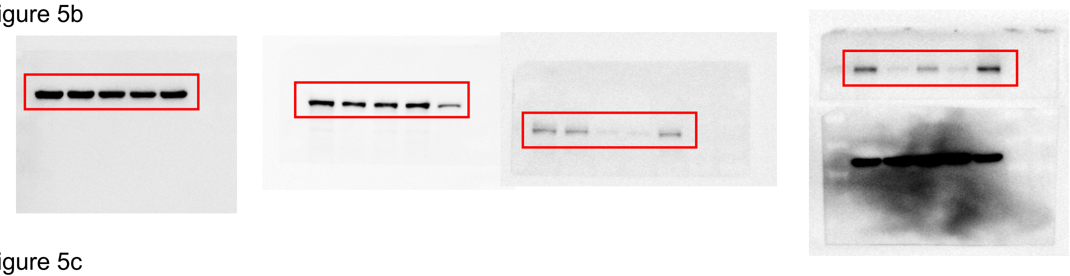

Figure 5c

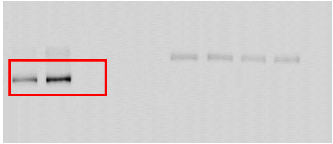

Figure 5e

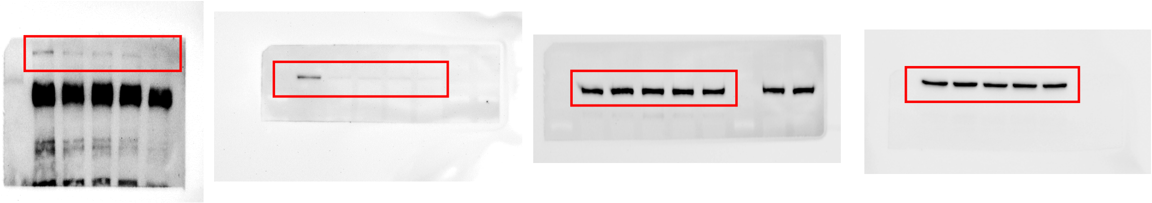

Figure 5f

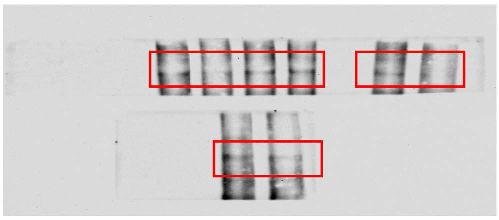

Figure 5g

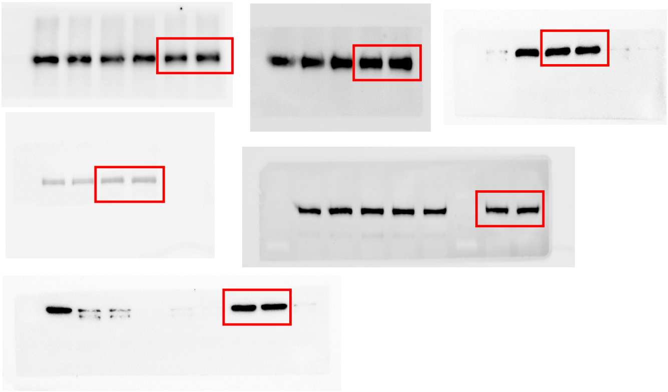

Figure 5h

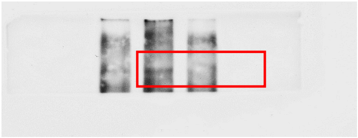

Figure 6a

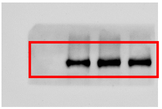

Figure 6c

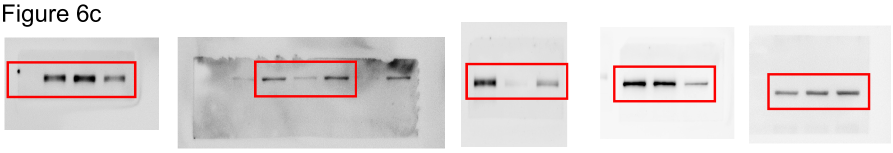

Suppl. Figure 8: Uncropped/raw gel images associated with Figures 5 and 6. Red boxes indicate region of cropped image.

Figure 7a

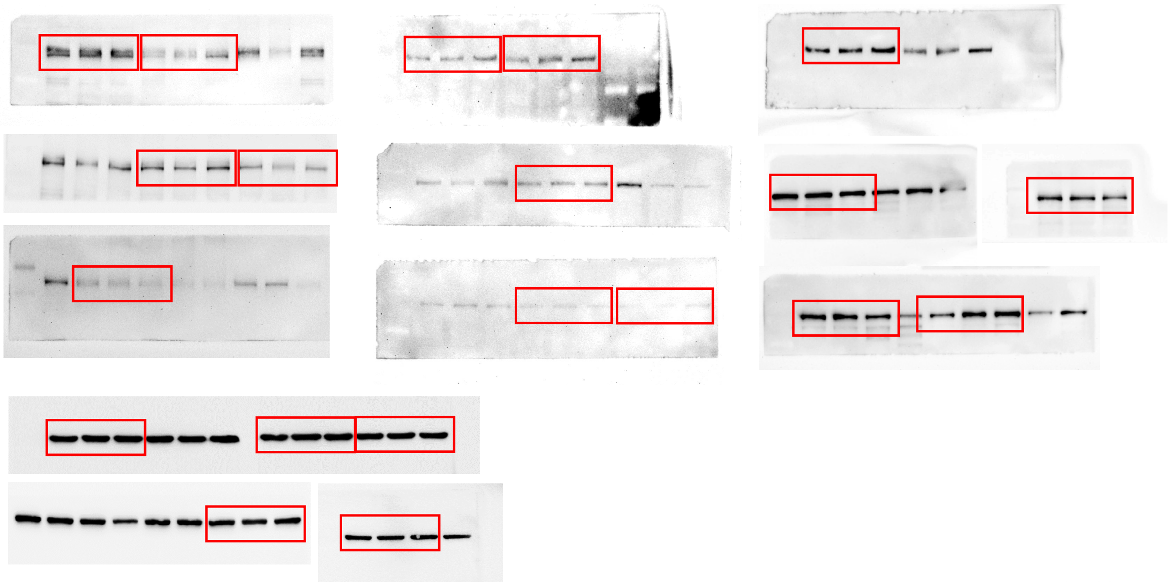

Figure 7i

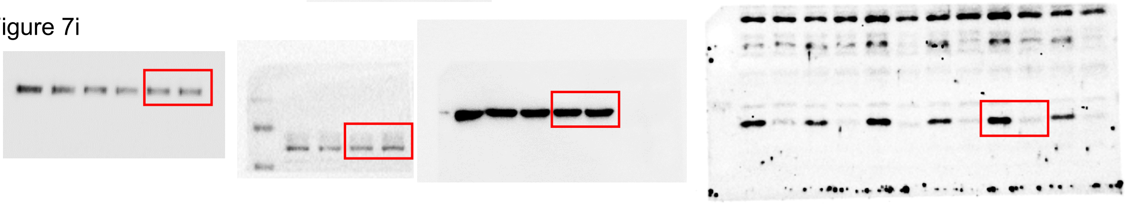

Suppl. Figure 3a

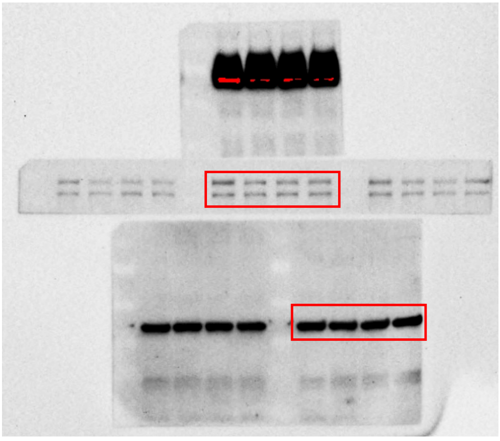

Suppl. Figure 4d

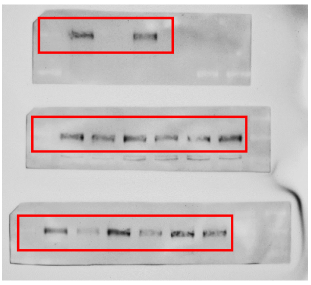

Suppl. Figure 9. Uncropped/raw gel images associated with Figures 7, Supplementary Figure 3, and Supplementary Figure 4. Red boxes indicate region of cropped image.
