# Supplementary material for: A proteomics approach to isolating neuropilin-dependent α5 integrin trafficking pathways: neuropilin 1 and 2 co-traffic α5 integrin through endosomal p120RasGAP to promote polarised fibronectin fibrillogenesis in endothelial cells
Source: Commun Biol. 2024 May 24;7:629. doi: 10.1038/s42003-024-06320-4 (PMC11126613; doi:10.1038/s42003-024-06320-4)
Supplement: Supplementary file 4 — Reporting Summary [file 42003_2024_6320_MOESM4_ESM.pdf]

nature portfolio

Corresponding author(s): Benwell, Robinson

Last updated by author(s): May 11, 2024

Reporting Summary

Nature Portfolio wishes to improve the reproducibility of the work that we publish. This form provides structure for consistency and transparency in reporting. For further information on Nature Portfolio policies, see our Editorial Policies and the Editorial Policy Checklist.

Statistics

For all statistical analyses, confirm that the following items are present in the figure legend, table legend, main text, or Methods section.

n/a	Confirmed	
☐	☒	The exact sample size (*n*) for each experimental group/condition, given as a discrete number and unit of measurement
☐	☒	A statement on whether measurements were taken from distinct samples or whether the same sample was measured repeatedly
☐	☒	The statistical test(s) used AND whether they are one- or two-sided<br>*Only common tests should be described solely by name; describe more complex techniques in the Methods section.*
☒	☐	A description of all covariates tested
☐	☒	A description of any assumptions or corrections, such as tests of normality and adjustment for multiple comparisons
☐	☒	A full description of the statistical parameters including central tendency (e.g. means) or other basic estimates (e.g. regression coefficient) AND variation (e.g. standard deviation) or associated estimates of uncertainty (e.g. confidence intervals)
☐	☒	For null hypothesis testing, the test statistic (e.g. *F*, *t*, *r*) with confidence intervals, effect sizes, degrees of freedom and *P* value noted<br>*Give P values as exact values whenever suitable.*
☒	☐	For Bayesian analysis, information on the choice of priors and Markov chain Monte Carlo settings
☒	☐	For hierarchical and complex designs, identification of the appropriate level for tests and full reporting of outcomes
☒	☐	Estimates of effect sizes (e.g. Cohen's *d*, Pearson's *r*), indicating how they were calculated

Our web collection on statistics for biologists contains articles on many of the points above.

Software and code

Policy information about availability of computer code

Data collection	N/A
Data analysis	N/A

For manuscripts utilizing custom algorithms or software that are central to the research but not yet described in published literature, software must be made available to editors and reviewers. We strongly encourage code deposition in a community repository (e.g. GitHub). See the Nature Portfolio guidelines for submitting code & software for further information.

Data

Policy information about availability of data

All manuscripts must include a data availability statement. This statement should provide the following information, where applicable:

- Accession codes, unique identifiers, or web links for publicly available datasets
- A description of any restrictions on data availability
- For clinical datasets or third party data, please ensure that the statement adheres to our policy

The raw data supporting the conclusions of this article will be made available by the authors, without undue reservation, to any qualified researcher.

Human research participants

Policy information about studies involving human research participants and Sex and Gender in Research.

Reporting on sex and gender	N/A
Population characteristics	N/A
Recruitment	N/A
Ethics oversight	N/A

Note that full information on the approval of the study protocol must also be provided in the manuscript.

Field-specific reporting

Please select the one below that is the best fit for your research. If you are not sure, read the appropriate sections before making your selection.

☒ Life sciences ☐ Behavioural & social sciences ☐ Ecological, evolutionary & environmental sciences

For a reference copy of the document with all sections, see nature.com/documents/nr-reporting-summary-flat.pdf

Life sciences study design

All studies must disclose on these points even when the disclosure is negative.

Sample size	For all animal studies a minimum sample size of n ≥ 3 mice was used to achieve a 95% power to detect a mean difference of 1 standard deviation with a significance of 0.05 using one-way ANOVA tests or Student's t-tests. Sample sizes were also contributed to by independent replicate experiments.
Data exclusions	No data exclusions were performed.
Replication	All attempts at replication were successful. All experiments were repeated in multiple independent experiments. Within the figure legends, the number of replicates is defined.
Randomization	All mice used in this study were weight and age matched. Cre-recombinase expression and therefore gene excision was determined post humane killing to ensure sample randomisation.
Blinding	No blinding was performed because the data acquired was obtained by using objective quantitative methods and researchers did not have expectations regarding the outcome.

Reporting for specific materials, systems and methods

We require information from authors about some types of materials, experimental systems and methods used in many studies. Here, indicate whether each material, system or method listed is relevant to your study. If you are not sure if a list item applies to your research, read the appropriate section before selecting a response.

Materials & experimental systems

n/a	Involved in the study	
☐	☒	Antibodies
☐	☒	Eukaryotic cell lines
☒	☐	Palaeontology and archaeology
☐	☒	Animals and other organisms
☒	☐	Clinical data
☒	☐	Dual use research of concern

Methods

n/a	Involved in the study	
☒	☐	ChIP-seq
☒	☐	Flow cytometry
☒	☐	MRI-based neuroimaging

Antibodies

Antibodies used	α5 integrin<br>CST: 4705S, RRID: AB_2233962, 1:1000<br>Validation criteria: https://www.cellsignal.com/products/primary-antibodies/integrin-a5-antibody/4705

Ab25251, RRID: AB_448738, 1:200
Validation criteria: https://www.abcam.com/products/primary-antibodies/integrin-alpha-5-antibody-5h10-27-mfr5-ab25251.html

β-actin
CST: 8457S, RRID: AB_10950489, 1:2000
Validation criteria: https://www.cellsignal.com/products/primary-antibodies/b-actin-d6a8-rabbit-mab/8457

NRP1
R&D: AF566, RRID: AB_355445, 1:100
Validation criteria: https://www.rndsystems.com/products/mouse-rat-neuropilin-1-antibody_af566

NRP2
SCB: sc-13117, RRID: AB_628044, 1:50
Validation criteria: https://www.scbt.com/p/neuropilin-2-antibody-c-9
CST: 3366, RRID: AB_2155250, 1:1000
Validation criteria: https://www.cellsignal.com/products/primary-antibodies/neuropilin-2-d39a5-xp-rabbit-mab/3366

Clathrin heavy chain-1
Ab21679, RRID: AB_2083165, 1:100
Validation criteria: https://www.abcam.com/products/primary-antibodies/clathrin-heavy-chain-antibody-ab21679.html

Dynamin-2
Ab3457, RRID: AB_2093679, 1:100
Validation criteria: https://www.abcam.com/products/primary-antibodies/dynamin-2-antibody-ab3457.html

EEA1
Ab2900, RRID: AB_2262056, 1:100
Validation criteria: https://www.abcam.com/products/primary-antibodies/eea1-antibody-early-endosome-marker-ab2900.html

Rab11
Ab3612, RRID: AB_10861613, 1:2000
Validation criteria: https://www.abcam.com/products/primary-antibodies/rab11b-antibody-ab3612.html

Rab4
Ab13252, RRID: AB_2269374, 1:100
Validation criteria: https://www.abcam.com/products/primary-antibodies/rab4-antibody-early-endosome-marker-ab13252.html

Rab7
CST: 17286, RRID: AB_1904103, 1:100
Validation criteria: https://www.cellsignal.com/products/antibody-conjugates/rab7-d95f2-xp-rabbit-mab-alexa-fluor-555-conjugate/17286

αV integrin
Inv: PA5-47096, RRID: AB_2609681, 1:200/1:1000
Validation criteria: https://www.thermofisher.com/antibody/product/CD51-Antibody-Polyclonal/PA5-47096

Paxillin
Ab32084, RRID: AB_779033, 1:100
Validation criteria: https://www.abcam.com/products/primary-antibodies/paxillin-antibody-y113-ab32084.html
CST: 2542, RRID: AB_10693603, 1:1000
Validation criteria: https://www.cellsignal.com/products/primary-antibodies/paxillin-antibody/2542

Tensin-1
NB: NBP1-84129, RRID: AB_11014791, 1:100
Validation criteria: https://www.novusbio.com/products/tensin-1-antibody_nbp1-84129

EDA-FN
Sigma: F6140, RRID: AB_476981, 1:400
Validation criteria: https://www.sigmaaldrich.com/GB/en/product/sigma/f6140

p120RasGAP
Ab2922, RRID: AB_303418, 1:100/1:1000
Validation criteria: https://www.abcam.com/products/primary-antibodies/rasa1-antibody-b4f8-ab2922.html

Rab21
SCB: sc-81917, RRID: AB_2253236, 1:100/1:1000
Validation criteria: https://www.scbt.com/p/rab-21-antibody-b16k

Dynein
Thermo: MA1-070, RRID: AB_2093668, 1:200
Validation criteria: https://www.thermofisher.com/antibody/product/Dynein-Antibody-clone-74-1-Monoclonal/MA1-070

FAK
CST: 3285, RRID: AB_2269034, 1:1000
Validation criteria: https://www.cellsignal.com/products/primary-antibodies/fak-antibody/3285

pFAKTyr397
CST: 8556, RRID: AB_10891442, 1:1000
Validation criteria: https://www.cellsignal.com/products/primary-antibodies/phospho-fak-tyr397-d20b1-rabbit-mab/8556

pFAKTyr407
Inv: 44-650G, RRID: AB_2533708, 1:1000/1:200
Validation criteria: https://www.thermofisher.com/antibody/product/Phospho-FAK-Tyr407-Antibody-Polyclonal/44-650G

GM130
NB: NBP2-53420, RRID: AB_2916095, 1:100
Validation criteria: https://www.novusbio.com/products/gm130-golga2-antibody_nbp2-53420

Collagen IV
Ab19808, RRID: AB_445160, 1:500
Validation criteria: https://www.abcam.com/products/primary-antibodies/collagen-iv-antibody-ab19808.html

Validation

All antibodies used are validated for detection of indicated proteins and specificity was confirmed by comparison to isotype control staining. All antibodies used in this study were commercially available by vendors and have been validated by the manufacturers. Validation and specific validation criteria can be found at the following vendor websites above.

Eukaryotic cell lines

Policy information about cell lines and Sex and Gender in Research

Cell line source(s)

Cell line: Immortalised mouse lung microvascular endothelial cells (IMMLECs)
Cells were isolated from the lungs of 6-8-week old mice, twice subject to magnetic activated cell sorting (MACS) using the endothelial cell marker endomucin, prior to immortalisation using polyoma-middle-T-antigen (PyMT).

Authentication

Endothelial identity was authenticated at multiple passage points by western blotting.

Mycoplasma contamination

All cell lines were tested negative for mycoplasma prior to their use.

Commonly misidentified lines
(See ICLAC register)

N/A

Animals and other research organisms

Policy information about studies involving animals; ARRIVE guidelines recommended for reporting animal research, and Sex and Gender in Research

Laboratory animals

In this study we used male and female mice that were either between 6-8 weeks of age (cell line generation) or 6 days of age (retina experiments).

Mouse: C57BL/6 Charles River: 027 (subsequently bred in house)
Mouse: Nrp1 fl/fl Jackson Laboratories JAX: 006707
Mouse: Nrp2 fl/fl Jackson Laboratories JAX: 006700
Mouse: alpha 5 integrin fl/fl provided by Richard Hynes (Massachusetts Institute of Technology, USA)
Mouse: Pdgfb.iCreERT2 provided by Marcus Fruttiger (UCL, London, UK)

Wild animals

No wild animals were used in this study.

Reporting on sex

Male and female mice were used.

Field-collected samples

No field-collected samples were used in this study.

Ethics oversight

All experiments were performed in accordance with UK home office regulations and the European Legal Framework for the Protection of Animals used for Scientific Purposes (European Directive 86/609/EEC). All experiments were approved by the Animal Welfare and Ethical Review Board (AWERB) committee at the University of East Anglia, UK.

Note that full information on the approval of the study protocol must also be provided in the manuscript.
